# Functional Dyspepsia, Anæmia, and Chlorosis, New Treatment of

**Published:** 1873-05

**Authors:** C. E. Brown-Sequard


### On a New Mode of Treatment of Functional Dyspepsia, Anæmia, and Chlorosis.

By C. E. Brown-Sequard, M. D.

In 1851 I had to treat a very bad case of dyspepsia, and succeeded to cure the patient by a plan of treatment which, I think, deserves attention. Since that time I have employed it with complete or partial success in a number of cases of dyspepsia, of chlorosis, of anæmia, and also as a means of ameliorating or curing nervous affections caused by gastric disturbances or poverty of blood. I could not say, as I have not kept notes in all the cases, how many times it has succeeded or failed. In a number of instances where failure occurred, I have found that the patients had not carefully followed the rules, and that the failure was, at least in a good measure, due to this lack of care. In two cases only some increase of flatulency and of acid eructations took place during three or four days, when the plan was given up. In a case of dropsy, attended with anæmia, dyspeptic pains were increased for a week, when the plan was abandoned. These are the only instances I remember in which some bad effect was produced by this plan, and this aggravation soon ceased.

The first patient I submitted to this plan was a scientific man, 34 years old, of strong constitution, but reduced from several causes to a lamentable state of health. For about eight years he had been working very hard, taking no exercise, and living almost all the time in a vitiated atmosphere. He slept very little, and usually passed 18 or even 19 hours a day writing, reading, or experimenting. His diet was miserable, and, with the object of avoiding the need of much food, he took a great deal of coffee. He gradually, though slowly, became exceedingly weak. His digestion, which had been very good all his life, before he began to work so much, had gradually become very bad. He suffered greatly from pyrosis, and a feeling of great distress, and gastric distention after each meal. Acid eructations and gas were frequently thrown up into his mouth, and when he did not vomit he found that his food remained on his stomach so long, that in the morning he frequently rejected things eaten the previous day. At last he had to give up work and stay in bed. But no improvement occurred from the rest he then had, or from various modes of treatment. His emaciation and weakness and dyspeptic symptoms increased, and his friends decided to have him removed to the country. But he was so weak that he had to be carried in a litter to the railway station. After a few days, finding that he had not improved, I decided to try a radical change of his alimentation, as regards the quantity of food to be taken at a time. Instead of *three* meals a day I made him take *sixty* or more. Every twelve or fifteen minutes he took two or three mouthfuls of solid food, chiefly meat and bread. He drank a little less than a wine-glass of Bordeaux wine and water every thirty or forty minutes. On the very first day this mode of alimentation was begun, his digestive troubles* disappeared, and within a week he was so well that he returned to Paris, not, however, to go to work again, as he had been rendered wiser, but to prepare to go to the seashore. He continued the same mode of alimentation for about three weeks, and then gradually diminished the number of his homœopathic meals, and increased the amount taken at each of them, until in about 8 or 10 days he came to eat only three times a day, and a full meal at each time. His strength during the first week had become almost as great as it ever had been previous to his illness. Since that time up to this moment his life has been one of great hardship, which he has borne remarkably well, and dyspepsia has only troubled him in a slight degree, rarely and for short periods.

In one case only besides the preceding have I seen as rapid a return to health. That was the case of a young lady, whom I saw last year at Jamaica Plain, in consultation with my learned friend, Dr. S. Cabot, of Boston. In the case of this lady there was this additional good effect to this hygienic treatment, that the bowels, which were very costive before, began to act pretty well almost at once.

The plan, as stated in the above case, consists in giving but very little of solid or fluid food or any kind of drink at a time, and to give these things at regular intervals of from ten to twenty or thirty minutes. All sorts of food may be taken in that way, but during the short period when such a trial is made, it is obvious that the fancies of patients are to be laid aside, and that nourishing food, such as roasted or broiled meat, and especially beef and mutton, eggs, well-baked bread, and milk, with butter and cheese, and a very moderate quantity of vegetables and fruit, ought to constitute the dietary of the patients we try to relieve. This plan should be pursued two or three weeks, after which the patient should gradually return to the ordinary system of eating three times a day.

It is hardly possible to give more detailed rules as regards this hygienic mode of treatment. On the one hand I have found few persons willing or able to follow it fully. On the other hand, many patients, especially those who have no dyspepsia, do not need to take so minute an amount of food at a time. Besides, it is certain that the quantity of food required varies notably in different persons. Prof. John C. Dalton states that the entire amount of food needed by a man in full health and taking free exercise is: of meat, 16 oz. av.; bread, 19 oz.; fat, $3\frac{1}{2}$ oz.; and of water, 52 fl. oz.; i. e., about $2\frac{1}{2}$ lbs. of solid food, and rather more

* One of the symptoms which had preceded the others — merycism, persisted, and has remained ever since, being now as before of daily occurrence.

than 3 pints of fluid. According to Dr. Edward Smith and other European hygienists, the amount of solid food and of water required each day is notably larger than that marked out by the able American physiologist I have named. My experience with the patients on whom I have tried the plan of feeding above mentioned, shows that the amount of solid food required by an adult is nearly always as follows: from 12 to 18 oz. of cooked meat, and from 18 to 24 oz. of bread. As regards the quantity of fluids I have allowed, it has always been notably less than the amount indicated by Dr. Dalton (3 pints), and by Dr. E. Smith ($4\frac{1}{2}$ to 5 pints).

I hardly need say that in carrying out the plan I propose, attention must be paid to three points: 1st, the liking and the disliking of certain things by the patient; 2d, the importance of variety in food; 3d, the digestibility of certain things compared with others, digestibility which varies immensely in different patients. When I found that there was no disgust for a meat and bread diet, I ordered that roasted beef or mutton, with bread, be the almost only kinds of solid food taken. But most patients were either soon disgusted with this diet, or refused even to try it. Having ascertained this, I allowed the selection by each patient of his own dietary, insisting, however, that the quantity of cooked meat should be at least 12 oz. a day. The most varied diet as regards the kinds of food can be followed, however, under this plan as well as when one has only two or three meals a day. The only absolutely essential points are that the amount of food taken every 10, 15, 20 or 30 minutes be very small (from two to four mouthfuls), and that the quantity of solid food in a day be from 32 to 40 oz., or a little less when, instead of water, the patient drinks beef-tea or milk.

I will not enter into long explanations to show how a marked benefit or a cure can be obtained in functional dyspepsia, in anæmia, and other affections by this mode of alimentation. I will simply say that the facts I have observed agree with the view that we are naturally organized, like most if not all animals, to eat very frequeutly, and not, as we do, two, three, or four times a day. It seems certain from the facts I have observed that functional dyspepsia, when once it has begun (never mind by what cause), is kept up and increased by distention of the walls of the stomach. This fact is already well known, and physicians generally recommend that the quantity of liquid taken be very small, and that the solid food be as nourishing as possible, so that its bulk may be reduced, with the view of avoiding great dilatation by the fluid and solid substances introduced in the gastric pouch. But although deriving some benefit from this diminution of distention, many patients continue to suffer who might be benefited or cured by the plan I propose.

It may be asked if there is no danger that distention of the stomach, by a full ordinary meal, after a patient has followed for two, three, or four weeks the plan I propose, would not be more difficult and a source of greater trouble than before that organ had been allowed to contract considerably during the time this plan has been pursued. Facts answer this question in a way that leaves no doubt. There has never been in the cases I have attended the least trace of an increased trouble due to that cause. Even those patients who have not derived benefit from my plan of alimentation, and among them two who had while following it more acidity and flatulency, have, at any rate, had no increased trouble after having given it up. It is probable that the good obtained from this plan in dyspeptic patients depends at first on the rest given to the irritated stomach, and subsequently on a great amelioration in the quality of the gastric juice.

In anæmia and chlorosis, not complicated with dyspepsia, the advantage of this plan lies in the rapidity of formation of blood from the notably increased amount of food that the patient can digest.

I have made but very few trials — and incomplete ones — of this plan in cases of organic affections of the stomach. I cannot but think, however, that it deserves being tried in most of such cases.

Against the obstinate vomiting of pregnancy this plan has already been employed successfully by a number of physicians, and once by myself in a case in which many modes of medical treatment had failed. — *Archives of Scientific and Pract. Medicine.*

